# Type I conventional dendritic cells relate to disease severity in virus‐induced asthma exacerbations

**DOI:** 10.1111/cea.14116

**Published:** 2022-03-03

**Authors:** Aoife Cameron, Jaideep Dhariwal, Nadine Upton, Ismael Ranz Jimenez, Malte Paulsen, Ernie Wong, Maria‐Belen Trujillo‐Torralbo, Ajerico del Rosario, David J. Jackson, Michael R. Edwards, Sebastian L. Johnston, Ross P. Walton, the MRC‐GSK Strategic Alliance Consortium

**Affiliations:** ^1^ National Heart and Lung Institute London UK; ^2^ MRC Asthma UK Centre in Allergic Mechanisms of Asthma London UK; ^3^ School of Immunology & Microbial Sciences King’s College London London UK; ^4^ St. Mary’s Flow Cytometry Core Facility London UK; ^5^ Novo Nordisk Foundation Center for Stem Cell Medicine Faculty of Health and Medical Sciences University of Copenhagen Copenhagen Denmark; ^6^ Guy's and St Thomas’ NHS Trust London UK

**Keywords:** atopy, DC, rhinovirus

## Abstract

**Rationale:**

Rhinoviruses are the major precipitant of asthma exacerbations and individuals with asthma experience more severe/prolonged rhinovirus infections. Concurrent viral infection and allergen exposure synergistically increase exacerbation risk. Although dendritic cells orchestrate immune responses to both virus and allergen, little is known about their role in viral asthma exacerbations.

**Objectives:**

To characterize dendritic cell populations present in the lower airways, and to assess whether their numbers are altered in asthma compared to healthy subjects prior to infection and during rhinovirus‐16 infection.

**Methods:**

Moderately‐severe atopic asthmatic patients and healthy controls were experimentally infected with rhinovirus‐16. Bronchoalveolar lavage was collected at baseline, day 3 and day 8 post infection and dendritic cells isolated using fluorescence activated cell sorting.

**Measurements and Main Results:**

Numbers of type I conventional dendritic cells, which cross prime CD8^+^ T helper cells and produce innate interferons, were significantly reduced in the lower airways of asthma patients compared to healthy controls at baseline. This reduction was associated serum IgE at baseline and with reduced numbers of CD8^+^ T helper cells and with increased viral replication, airway eosinophils and reduced lung function during infection. IgE receptor expression on lower airway plasmacytoid dendritic cells was significantly increased in asthma, consistent with a reduced capacity to produce innate interferons.

**Conclusions:**

Reduced numbers of anti‐viral type I conventional dendritic cells in asthma are associated with adverse outcomes during rhinovirus infection. This, with increased FcεR1α expression on lower airway plasmacytoid DCs could mediate the more permissive respiratory viral infection observed in asthma patients.

**Scientific knowledge on the subject**

People with asthma experience more severe and more prolonged rhinovirus infections, which are the major precipitant of asthma exacerbations. Dendritic cells (DCs) orchestrate both innate and adaptive responses to virus, however, there has been only limited characterization of human airway DC populations. We have very little information on whether DCs are differentially regulated in asthma, and no prior knowledge as to the kinetics of their recruitment to the airways during viral exacerbations of asthma.

**What this study adds to the field**

We present the novel observation that baseline numbers of type I conventional (c)DCs are reduced in the lower airways of people with asthma compared to healthy controls. Reduced numbers of type I cDCs were associated with greater baseline total and house dust mite‐specific serum IgE, as well as to adverse outcomes during infection including increased viral replication, greater falls in lung function, increased numbers of eosinophils, and reduced CD8^+^ T cell airway numbers.

The increased expression of IgE receptor FcεR1α on lower airway plasmacytoid DCs we demonstrate herein, may be indicative of a reduced ability to induce innate IFNs in response to virus infection.

**Key messages**

- Reduced baseline type I conventional dendritic cell (cDC) numbers in the lower airways of asthma patients compared to healthy controls.
- This was associated with greater baseline serum IgE concentrations and adverse outcomes during rhinovirus infection.
- There was increased expression of IgE receptor FcεR1α on lower airway plasmacytoid DCs in asthma patients compared to healthy controls.

## 1 INTRODUCTION

Respiratory viruses, especially rhinoviruses (RV), are the major precipitant of asthma exacerbations.^1^, ^2^ Concurrent viral infection and allergen exposure synergistically increase risk of asthma exacerbation,^3^, ^4^, ^5^ with individuals with asthma experiencing more severe and more prolonged rhinovirus infections.^6^, ^7^ This increased disease may be due, at least in part, to impaired innate interferon (IFN) responses identified in airway cells from asthma patients.^8^, ^9^, ^10^ T helper type 2 (Th2) cytokines, which along with pro‐type 2 mediators IL‐25 and IL‐33, are induced in the lower airways of asthma patients during experimental RV‐16 infection^7^, ^11^, ^12^, ^13^ and have been shown to impair innate IFN responses.^14^, ^15^ A recent study used machine learning to cluster asthma characteristics based on immunophenotypes and demonstrated that children with the lowest *ex vivo* IFN responses to RV not only had a high risk of allergic sensitization but were at the greatest risk of hospitalization due to lower respiratory tract infections.^16^ These studies together emphasize the importance of understanding the interplay between type 2 and anti‐viral responses in viral asthma exacerbations.

The immune response to both virus and allergen is mediated by dendritic cells (DCs) which are airways sentinels which sample the lumen, sensing and interpreting signals to orchestrate downstream immune responses. Human DCs are grouped into conventional (c)DCs, the major antigen‐presenting subset and plasmacytoid (p)DCs, which are potent producers of type I IFNs following viral recognition.^17^ Human cDCs are further subtyped into type I and type II cDCs. Type II cDCs present antigen to CD4^+^ T cells and following viral recognition, prime Th1 cells. Human type I cDCs are reported to cross‐present viral antigen to CD8^+^ T cells,^18^ polarize Th1 cells^19^, ^20^ and produce IFN‐β and IFN‐λ.^21^

Most of our understanding of human DCs comes from studies in peripheral blood. Blood type II cDCs from asthma patients have an increased Th2 priming following allergen exposure, and reduced capacity to prime Th1 cells following LPS stimulation.^22^ Blood pDCs from asthma patients have impaired type I and type III IFN responses to viral infections,^23^ with crosslinking and signalling via the high‐affinity IgE receptor FcεRIα implicated as a mechanism.^24^, ^25^, ^26^ FcεRIα expression has been assessed on blood DCs in asthma,^23^, ^24^ however, differences in receptor expression between asthmatic and healthy subjects have not been characterized on lower airway DC populations.

Whilst a wealth of understanding has been gained from complex mouse models interrogating the role of DCs in airway disease,^27^ the limited studies that assessed airway DCs in asthma did not include comparisons with healthy volunteers, instead comparisons were made with lung tissue from non‐atopic patients undergoing lung resection due to cancer or other illness, with close proximity to diseased tissue likely to affect the phenotype and function of DCs.^28^, ^29^

DCs have not been characterized in human airways during a respiratory viral infection. In this study we characterize the DC subsets present in the lower airways of asthma patients and healthy controls using flow cytometry at baseline, and during experimental rhinovirus infection. Some of the results of these studies have been previously reported in the form of abstracts.^30^, ^31^, ^32^

## 2 METHODS

### 2.1 Ethics and consent

This study was approved by the London Bridge Research Ethics Committee (reference 12/LO/1278) and was carried out in accordance with the Declaration of Helsinki and Good Clinical Practice Guidelines. Informed consent was obtained from all subjects prior to their participation.

### 2.2 Study participants

Non‐smoking volunteers with moderately‐severe asthma that was not well‐controlled (with an Asthma Control Questionnaire (ACQ) score greater than 0.75^33^) and non‐smoking, non‐atopic, healthy volunteers were recruited as described in *Dhariwal* et al. (2021). Asthma patients were on treatment with either inhaled corticosteroids (ICS) or a combination inhaler (long acting beta agonist (LABA) + ICS), had objective airway hyperresponsiveness (AHR) with a (PC)_20_ histamine <8 µg/ml, and evidence of atopy on skin prick testing (≥1 positive skin prick test on a panel of 10 aeroallergens). Table 1 reports clinical characteristics of both subject groups, full inclusion and exclusion criteria are displayed in the online supplement (See Table S1) *Dhariwal* et al. (2021).

**TABLE 1:** Baseline characteristics of confirmed infected study subjects

	Healthy (*n *= 12)	Asthma (*n *= 11)	*p*‐value
Age (years)	22 (20, 31.25)	26 (20, 42)	ns
Gender	8M:4F	7M:4F	
Baseline FEV_1_ (L)	97.5 (91, 106)	87 (78, 96)	.032
PC_20_ (mg/ml)	>8	0.48 (0.06, 2)	–
ICS daily dose µg	0	400 (400, 1000)	–
No. of positive SPTs^π^ (out of 10)	0	4 (2, 6)	–
IgE (IU/ml)	27.7 (10.05, 58.33)	200.5* (151.5, 351.5)	<.001
ACQ	N/A	1.5 (1.2, 1.66)	–

Note

Data is displayed as median (25th, 75th percentile) values and statistical analysis was carried out using Mann Whitney *U* tests.

Abbreviations: ACQ, asthma control questionnaire; FEV_1_, forced expiratory volume in 1 s; PC_20_, concentration of histamine required to reduce FEV_1_ by 20%; ICS, inhaled corticosteroid; SPT, skin prick test; N/A, not applicable; NS, non‐significant.

*n = 10 asthmatic patients. **^π^** Allergens tested: five grass pollen mix, cat dander, Aspergillus fumigatus, *Alternaria alternata*, 3 tree pollen mix, house dust mite, mugwort, dog hair, *Clasdosporium herbarum*, birch pollen (ALK Abello).

### 2.3 Study design

**FIGURE 1:**
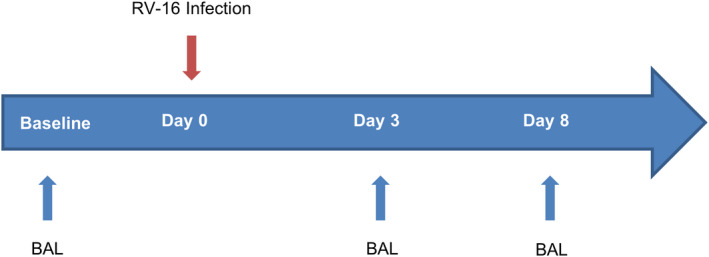
Overview of RV‐16 challenge clinical protocol. Atopic asthmatic subjects and healthy controls were experimentally infected intranasally with RV‐16 on day 0. Dendritic cells were isolated from BAL collected during bronchoscopy, at baseline (approximately day −14) and day 3 and day 8 post‐infection. Spirometry and symptom diaries and additional upper airway sampling occurred throughout the duration of the study from baseline to 6 weeks (For further details see Table S2 and Dhariwal et al. [2021]). Abbreviations: BAL, bronchoalveolar lavage

Subjects were experimentally infected on day (D)0 with rhinovirus (RV‐A16) intra‐nasally at 100 tissue culture 50% infective dose (TCID_50_) as previously described.^7^ Subjects underwent bronchoscopies at baseline (approximately day −14) and on D3 and D8 post infection (Figure 1). Virus load was determined in nasal lavage and induced sputum samples to confirm infection as described in *Dhariwal* et al. (2021). Spirometry was performed at baseline, D3 and D8, and daily symptom scores were assessed at home (see Table S2 for details of data and sample collection). The clinical outcomes of experimental RV‐16 infection are described in *Dhariwal* et al. (2021).

### 2.4 Bronchoalveolar lavage processing and flow cytometry sorting and analysis

Bronchoalveolar lavage (BAL) cells were processed immediately following bronchoscopy and a primary flow cytometry sort was carried out to enrich DCs and T cells, in addition to other cell types of interest, as described in in *Dhariwal* et al. (2021). Following the primary sort, HLA‐DR^+^ lineage^−^ cells were stained using the BAL DC antibody panel (Table S3). Live, single, HLA‐DR^+^ lineage^−^ were sorted into type I cDCs (CD11c^+^ BDCA3^+^), type II cDCs (CD11c^+^ BDCA1^+^) and pDCs (CD123^+^ BDCA2^+^ BDCA4^+^), see Figure S1 for the gating strategies used. The number of DCs/ml BAL were calculated for each patient by multiplying their percentage of total live cells, following flow cytometric analysis, by the total number of HLA‐DR^+^ lineage^−^ cells stained. The expression of FcεRIα was measured on lower airway DC populations using flow cytometry (in a subset of eight asthma patients at baseline and seven during infection, and in four healthy controls at baseline and during infection), and the percentage of each DC subset expressing FcεRIα was determined (positive staining was determined using fluorescence minus one (FMO) controls). Flow cytometry isolated CD3^+^ cells were stained with the T cell panel (Table S3) and acquired using a Becton Dickinson FACS Aria II. Frequencies of CD8^+^ T cells were determined using the following gating strategy: single, live, CD3^+^, CD8^+^ CD4^−^ cells. BAL eosinophils were determined using differential cell counts on cytospins.^11^

### 2.5 Serum IgE measurement using ImmunoCAP

Total IgE levels were measured in serum using a total IgE ImmunoCAP system on a Phadia 100 reader (Thermo Fisher). Levels of Der p1 and Der p2‐specific IgE in human serum were measured using the ImmunoCAP Der p1 and Der p2‐specific IgE protocols (Thermo Fisher).

### 2.6 Statistical analysis

Statistical analysis was performed using Prism 6 (GraphPad Software). Data are presented as median (interquartile range), Kruskal–Wallis tests were first carried out, and where statistical significance was observed followed by either Wilcoxon's signed rank test to determine statistical differences within groups or Mann–Whitney *U* tests to determine differences between groups. Correlations were investigated using Spearman's correlation coefficient. Differences were considered statistically significant at *p* values <.05. All *p* values are two‐sided.

## 3 RESULTS

Representative flow cytometric plots of conventional (left hand panel) and plasmacytoid (right hand panel) lower airway DCs from one asthma patient and one healthy subject at baseline, and D3 and D8 post RV‐16 infection are displayed in Figure S2.

### 3.1 Enumeration of DC populations in the lower airways at baseline

Type II cDCs were the most numerous DC population in the lower airways. In healthy subjects numbers of type II cDCs (28.95 [9.07, 57.55]) were significantly elevated in the lower airways compared to both type I cDCs (*p* = .0001) (3.47 [1.72, 5.90]) and pDCs (*p* = .013) (3.26 [1.26, 9.03]). In asthma subjects, numbers of type II cDCs (17.17 [5.39, 36.87]) were significantly increased in the lower airways compared to pDCs (*p* = .0001) (2.38 [1.00, 3.29]).

**FIGURE 2:**
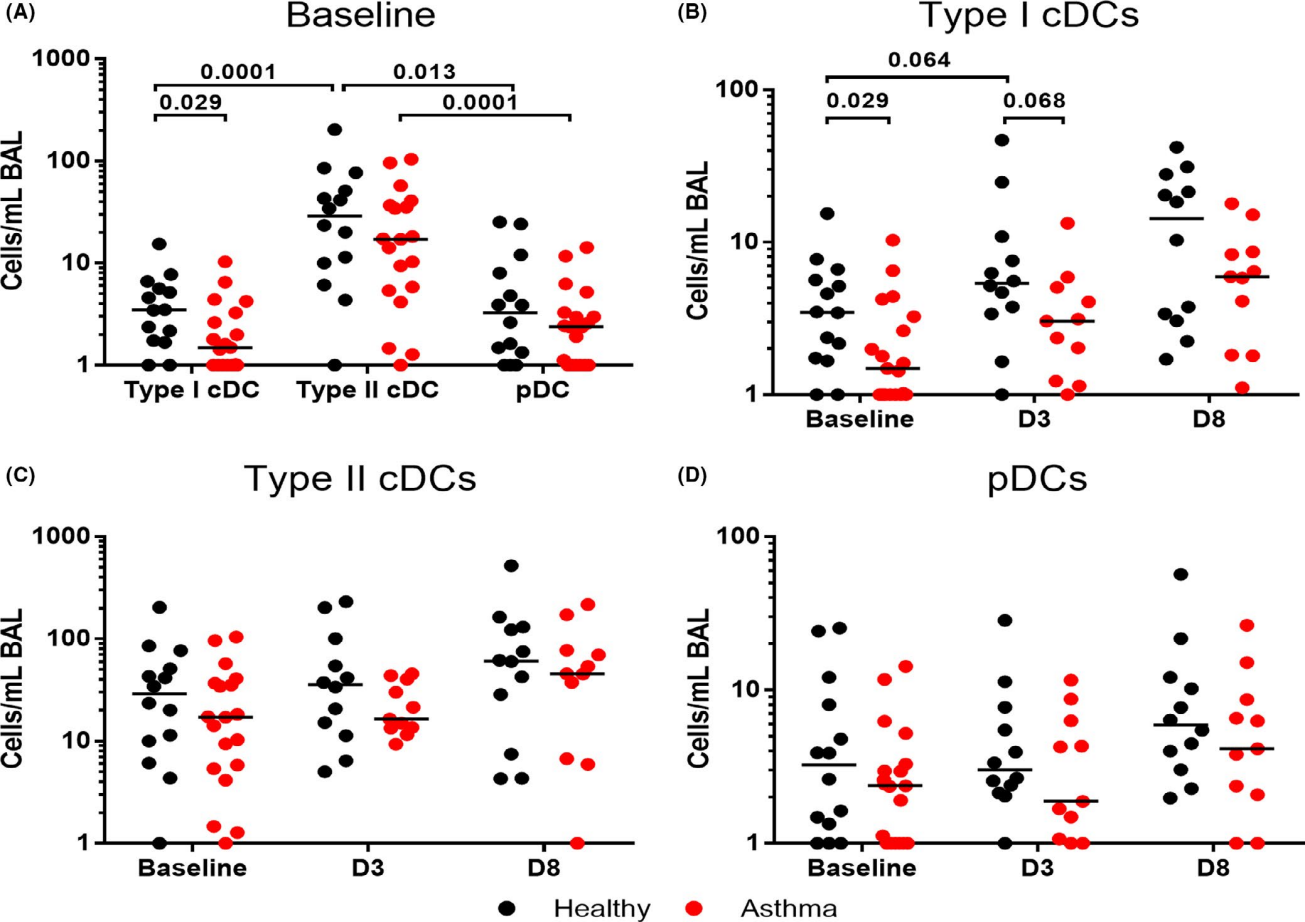
Assessment of DC populations in the lower airways at baseline and following experimental RV‐16 infection. BAL was collected at baseline, and on D3 and D8 following experimental RV‐16 infection in atopic asthmatic and healthy control subjects. DC populations are displayed at baseline (A) and changes from baseline to D3 and D8 post RV‐16 infection, for type I cDCs (B), type II cDCs (C) and pDCs (D). Data expressed as number of cells/ml BAL retrieved. Bars represent median values. Kruskal‐Wallace tests were used (not shown if not significant), prior to further statistical analysis using Wilcoxon matched pairs or Mann Whitney *U* test. *p* values <.1 are displayed. Baseline *N* = 14 healthy controls (black) and *N* = 19 asthma patients (red). D3 and D8 *N* = 12 confirmed RV‐16 infected healthy controls and *N* = 11 confirmed infected atopic asthmatic patients

Numbers of type I cDCs/ml BAL were significantly reduced in asthma patients (1.49 [0.56, 3.26]) compared to healthy controls (3.47 [1.72, 5.90]) at baseline (*p* = .029). Numbers of type II cDCs and pDCs at baseline tended to be lower in asthma patients compared to healthy controls but there were no statistically significant differences observed in numbers of type II cDCs and pDCs between subject groups (Figure 2A).

### 3.2 Enumeration of DC populations in the lower airways during experimental RV‐16 infection

There were no statistically significant changes in DC populations within the airways during rhinovirus infection, in either healthy subjects or asthma patients, (Figure 2B–D). All DC subtypes tended to increase from baseline during infection, on both D3 and D8, with greatest numbers being present at D8; however, the closest these changes came to achieving statistical significance was the trend towards an increase in numbers of type I cDCs/ml BAL in the lower airways of healthy subjects from baseline (3.47 [1.72, 5.90]) to D3 (5.38 [3.49, 10.07]) (*p* = .064).

Following infection, the trends towards reduced numbers of type II cDCs and pDCs observed at baseline in asthma patients compared to healthy controls remained, but the closest these differences came to achieving statistical significance was the trend towards reduced type I cDCs/ml BAL in asthma patients (3.04 [1.23, 5.06]) compared to healthy controls (5.38 [3.49, 10.07]) on D3 post‐infection (*p* = .068) (Figure 2B).

### 3.3 Reduction of airway type I cDCs is associated with house dust mite‐specific IgE

**FIGURE 3:**
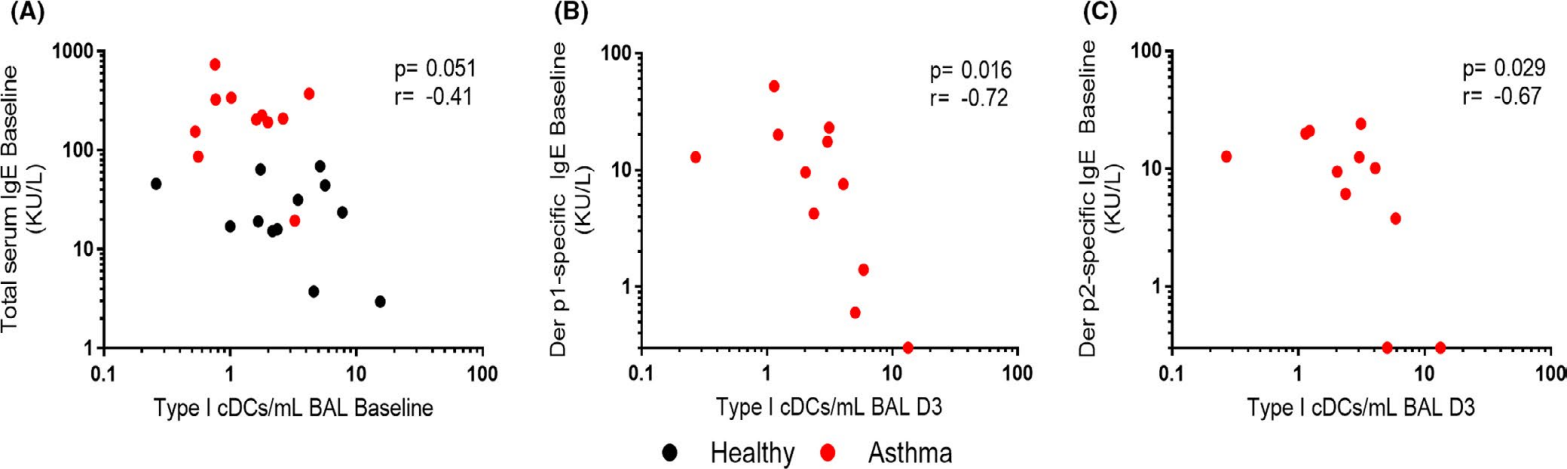
Numbers of type I cDCs in the lower airways are associated with atopy. Numbers of type I cDCs in BAL at baseline were correlated with baseline total serum IgE (A), and numbers of type I cDCs in BAL at D3 were correlated with baseline levels of both Der p1 (B) and Der p2‐specific IgE (C) using Spearman's correlation coefficient. Der p1 and Der p2 values below the assay lower limit of detection (0.3 KU/L) are displayed on the axis. Abbreviations: BAL, bronchoalveolar lavage. *N* = 12 confirmed RV‐16 infected non‐atopic healthy controls and *N* = 11 confirmed infected atopic asthmatic patients (all of whom were skin‐prick test positive for house dust mite)

We next sought to determine whether there was evidence to suggest that impaired DC numbers in asthma patients were associated with their atopic status. We identified a trend towards a negative correlation between the numbers of baseline type I cDCs in BAL and total serum IgE at baseline (*p* = .051, *r* = −.41) (Figure 3A and Table S4), and a significant relationship between type I cDCs in BAL at D3 and baseline levels of both Der p1‐ (*p *= .016, *r* = −.72) and Der p2‐specific serum IgE (*p *= .029, *r* = −.67) in asthma patients (Figure 3B,C).

### 3.4 Reduction of airway type I cDCs is associated with worse disease outcomes during infection

**FIGURE 4:**
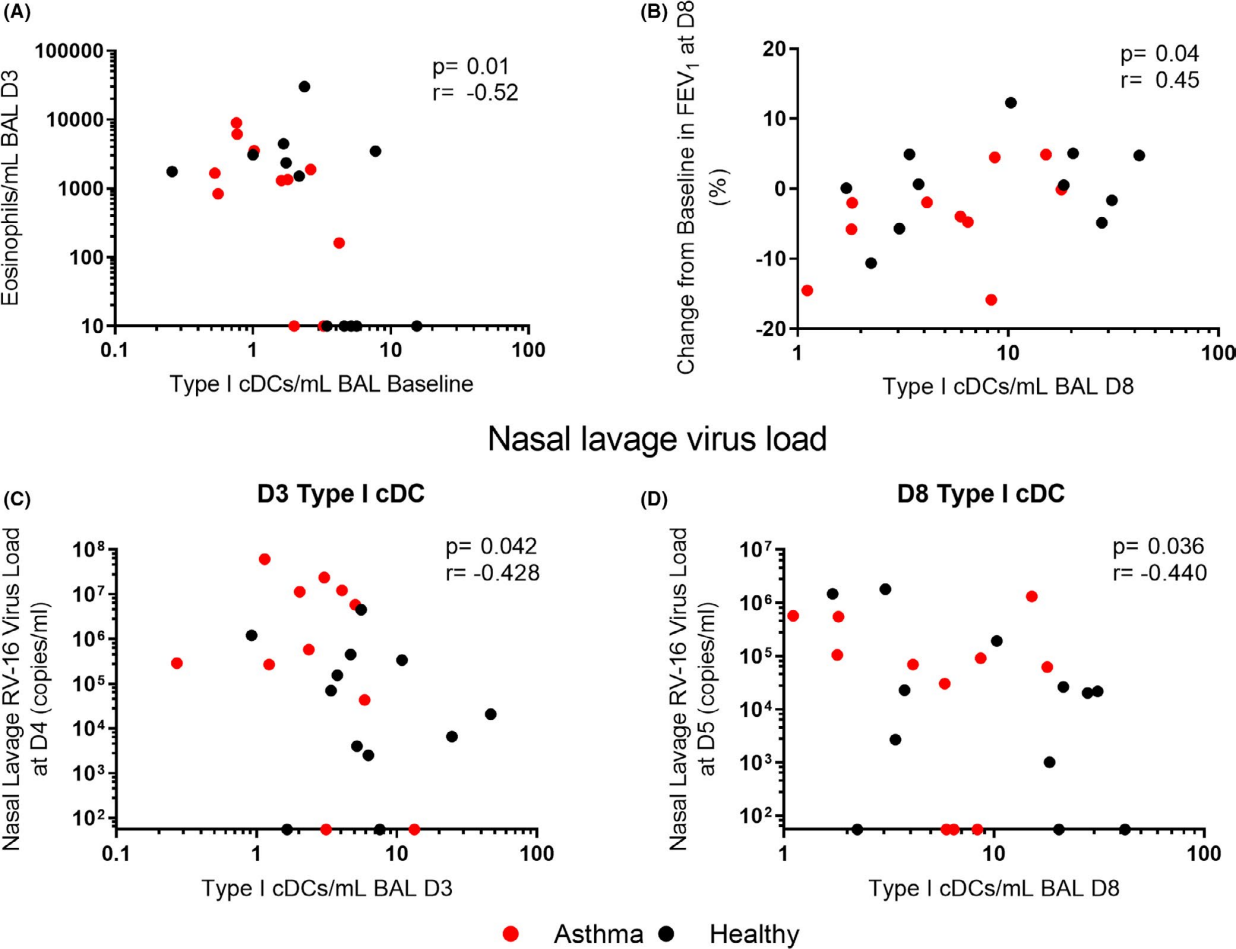
Greater numbers of type I cDCs in the lower airways are associated with better outcomes during infection. Numbers of type I cDCs in BAL were correlated with eosinophil numbers in the BAL at D3 post RV‐16 infection (A), change from baseline in FEV_1_ at D8 (B) and nasal lavage virus load at D4 (C) and D5 (D). Abbreviations: BAL, bronchoalveolar lavage, FEV_1_, forced expiratory volume in 1 s. *N* = 12 confirmed RV‐16 infected healthy controls and *N* = 11 confirmed infected atopic asthmatic patients, expect B where *N* = 11 healthy and *N* = 10 asthma. Nasal lavage virus load values below the assay lower limit of detection (55 copies/ml) are displayed on the axis

To determine whether the reduced numbers of type I cDCs identified in asthma patients at baseline were associated with clinical outcomes during infection, we performed correlation analyses with clinical parameters. We observed a negative correlation between numbers of type I cDCs in BAL at baseline and numbers of eosinophils in the BAL on D3 of infection (*p *= .01, *r* = −.52) (Figure 4A), indicating that reduced numbers of type I cDCs at baseline were associated with enhanced type 2 airways inflammation in response to infection. We also identified a positive correlation between the numbers of type I cDCs in BAL at D8 and change from baseline lung function at D8 post infection (*p *= .04, *r* = .45) (Figure 4B), indicating that greater numbers of type I cDCs at D8 were associated with protection from reductions in lung function in response to infection. Type I cDC numbers in BAL at D3 post‐infection were also inversely associated with nasal lavage virus load on D4 post‐infection in subjects (*p *= .042, *r* = −.428) (Figure 4C), and type I cDC numbers in BAL at D8 post‐infection were inversely associated with nasal lavage virus load on D5 post‐infection (*p *= .036, *r* = −.440) (Figure 4D). Correlations for the healthy control group and the asthma group when analysed separately are shown in Table S4.

### 3.5 Airway type I cDCs are associated with CD8^+^ T cell numbers during infection

**FIGURE 5:**
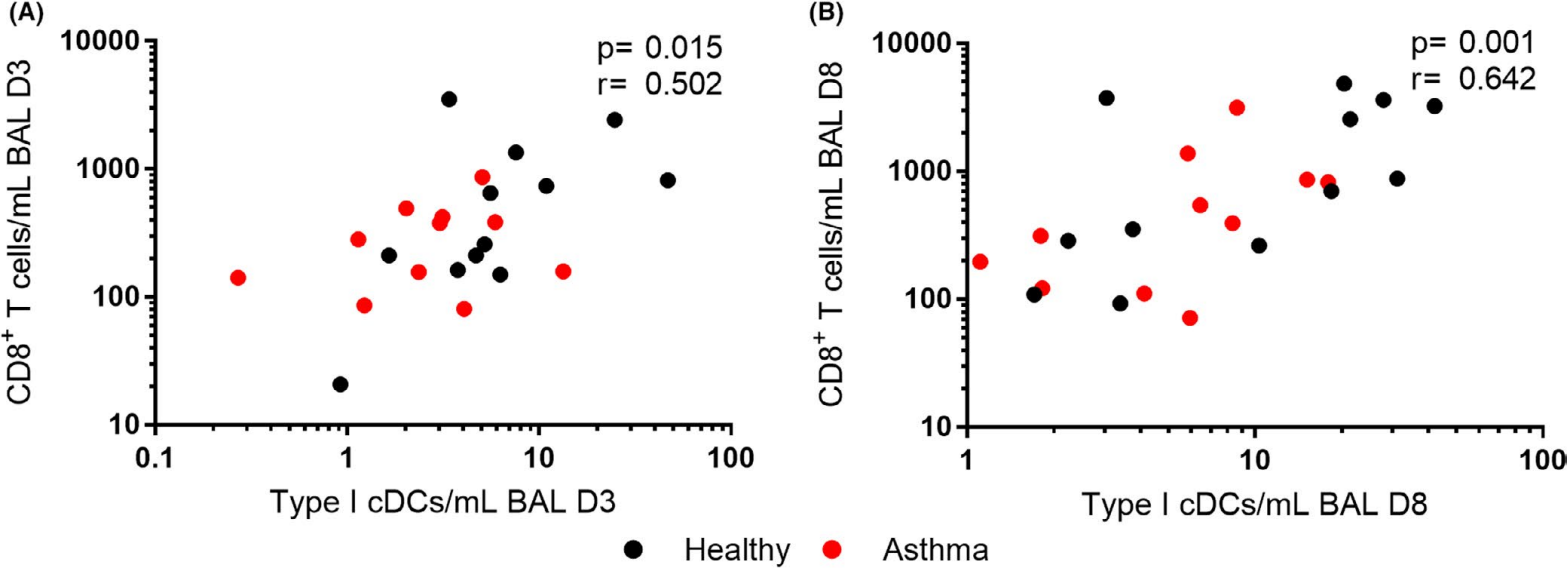
Numbers of type I cDCs in the lower airways are associated with CD8^+^ T cell recruitment. Numbers of type I cDCs in BAL were correlated with CD8^+^ T cell numbers in BAL at D3 (A) and D8 (B) post‐infection using Spearman's correlation coefficient. Abbreviations: BAL, bronchoalveolar lavage. *N* = 12 confirmed RV‐16 infected healthy controls and *N* = 11 confirmed infected atopic asthmatic patients

Since type I cDCs are reported to cross‐present viral antigens to CD8^+^ T cells^19^ we also assessed relationships between these two cell types during these RV infections and observed positive correlations between these cell populations in BAL at both D3 (*p* = .015, *r *= .502) and D8 post‐infection (*p *= .001, *r *= .642) (Figure 5A,B).

### 3.6 Increased FcεRIα expression on lower airway pDCs from asthma patients

Expression of the high‐affinity IgE receptor FcεRIα on peripheral blood pDCs has previously been shown be increased in asthma, and to inversely correlate with their production of IFN‐α in response to influenza virus infection.^24^ Increased FcεRIα expression has also been demonstrated on blood type II cDCs and pDCs from children with asthma compared to healthy children,^23^ however differences in FcεRIα expression have not been assessed on lower airway DC populations or characterized during a viral infection *in vivo*.

**FIGURE 6:**
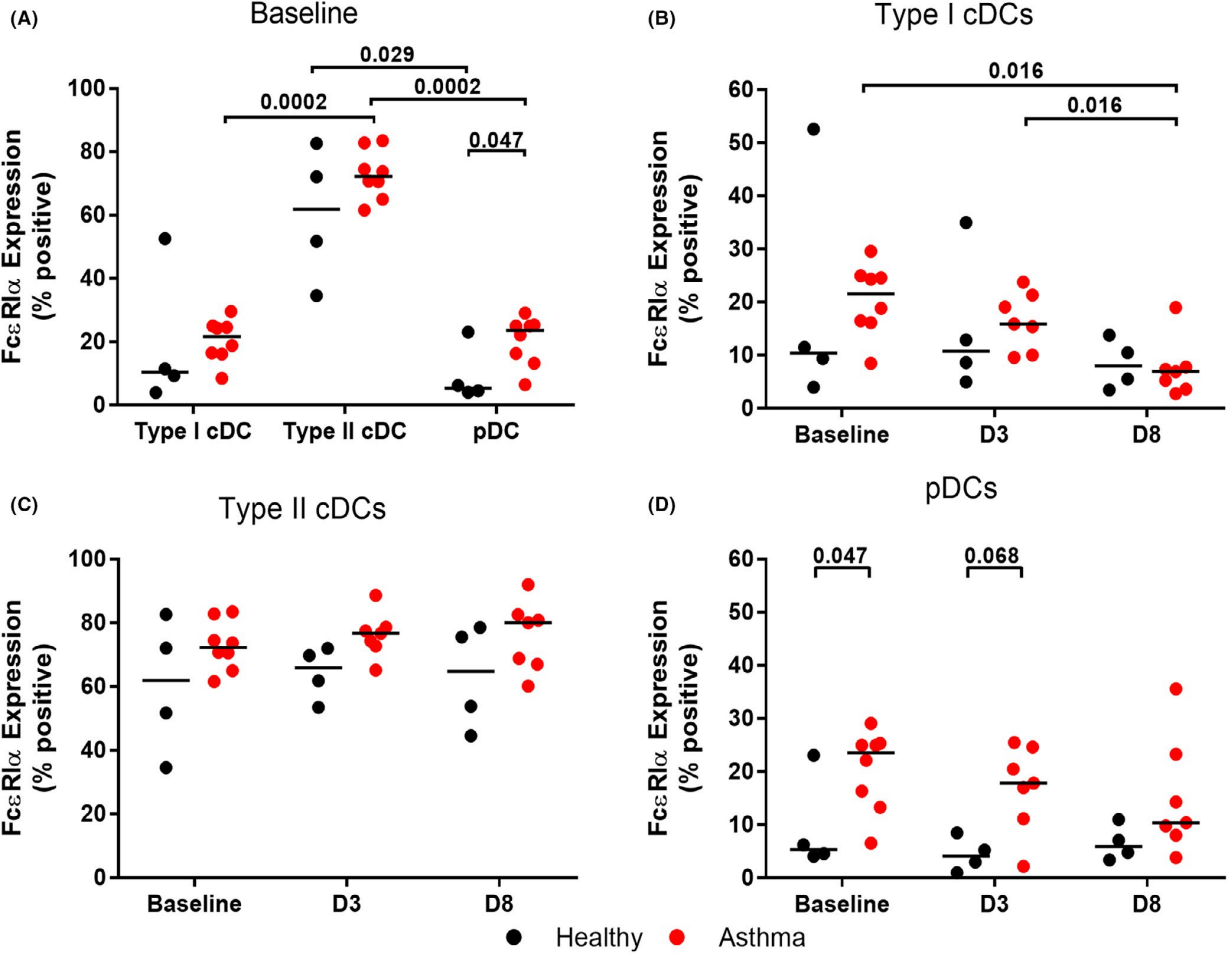
Analysis of FcεRIα expression in lower airway DCs. BAL was collected at baseline, and on D3 and D8 following experimental RV‐16 infection in atopic asthma patients and healthy control subjects. The percentage of DC populations expressing FcεRIα, as determined by flow cytometry, are displayed at baseline (A) and changes from baseline to D3 and D8 post RV‐16 infection, for type I cDCs (B), type II cDCs (C) and pDCs (D). Bars represent median values. Kruskal‐Wallace tests were used (not shown if not significant), prior to further statistical analysis using Wilcoxon matched pairs or Mann Whitney *U* test. *p* values <.1 are displayed. Baseline *N* = 4 healthy controls (black) and *N* = 8 asthma patients (red). D3 and D8 *N* = 4 confirmed RV‐16 infected healthy controls and *N* = 7 confirmed infected atopic asthma patients

We identified the expression of FcεRIα on both cDC and pDC populations in the lower airways at baseline, with the highest expression observed on type II cDCs (Figure 6A). In asthma patients at baseline, the percentage of type II cDCs expressing FcεRIα (72.3 (66.4, 80.8) was significantly higher compared to the percentage of type I cDCs (21.65 [16.28, 24.9]) (*p *= .0002) and pDCs (23.6 [14.08, 25.3]) (*p *= .0002) expressing the receptor (Figure 6A). A similar increased percentage of type II cDCs expressing FcεRIα (61.95 [38.9, 80.05]) was seen compared to pDCs in healthy subjects (5.44 [4.26, 18.89) (*p *= .029) (Figure 6A).

We identified, for the first time, the expression of FcεRIα on type I cDCs. There was no significant difference in expression between asthma patients and healthy controls (Figure 6B). Expression was down‐regulated in asthma patients following infection, with expression at D8 (6.98 [3.66, 7.80]) significantly reduced compared to both baseline (*p *= .016) (21.25 [16.28, 24.90]) and D3 (*p *= .016) (15.9 [10.10, 21.4]) (Figure 6B).

We did not identify a significant difference in the expression of FcεRIα on type II cDCs between asthma patients and healthy controls at baseline or following infection, though numbers tended to be higher in asthma patients at each time point (Figure 6C). The percentage of lower airway pDCs expressing FcεRIα was significantly increased in asthma patients (23.6 [14.08, 25.3]) compared to healthy controls (5.44 [4.26 18.89]) at baseline (*p *= .047), consistent with the elevated expression observed in peripheral blood,^23^, ^24^ with a trend towards increased expression in asthma (17.9 [11.2, 24.7]) at D3 post infection compared to healthy controls (4.15 [0.76, 7.70]) (*p *= .068) (Figure 6D).

## 4 DISCUSSION

To date, there has been only limited characterization of human airway DC populations.^28^, ^29^, ^34^ We have very little information regarding whether DCs are differentially regulated in asthma, and no prior knowledge as to the kinetics of their recruitment during viral exacerbations of asthma. To gain further understanding, moderately‐severe atopic asthma patients and healthy controls underwent lower airway sampling at baseline and then on D3 and D8 following experimental RV‐16 infection, and DC subsets were characterized in detail using flow cytometry.

Herein, we present the novel observation that baseline numbers of type I cDCs are reduced in the lower airways of asthma patients compared to healthy controls. Due to their crucial role in the activation of cytotoxic T cells, via cross‐presentation of viral antigens from infected cells,^18^ we speculate this could impair a critical arm of the anti‐viral immune response and may leave asthma patients vulnerable to infection. Viral replication has been shown to be increased in asthma and associated with worse disease outcomes in previous experimental infection studies.^7^, ^11^, ^35^ Here, we demonstrate that reduced numbers of type I cDCs in the lower airways are associated with greater virus loads, indicative of worse outcomes. Patients with reduced numbers of type I cDCs during infection also suffered from worsened lung function, with greater falls in FEV_1_ compared to their baseline levels. As type I cDC numbers correlated significantly with numbers of CD8^+^ T cells in the BAL at both D3 and D8 post‐infection, we propose that the reduced activation of CD8^+^ T cells, as a consequence of impaired type I cDCs numbers, could be a potential mechanism for the increased severity of infection experienced by asthma patients. Further work is required to determine whether the cytotoxic function CD8^+^ T cells is also reduced.

A protective role for type I cDCs in asthma exacerbation pathogenesis is also supported by the significant inverse relationship observed between baseline numbers of type I cDCs and eosinophil numbers in the BAL on D3 post‐infection. Eosinophils are recognized as a cell type important in asthma exacerbation pathogenesis, as higher levels of eosinophil cationic protein (ECP) concentrations in sputum in asthma exacerbations were associated with a longer hospital stays,^36^ and treatments reducing eosinophil numbers in asthma, such as anti‐IL‐5 therapeutics mepolizumab and benralizumab have been shown to reduce exacerbation frequency.^37^, ^38^

An increasing body of evidence suggests a synergistic relationship between viral infection and allergen exposure in sensitized individuals, which results in prolonged infections and increased exacerbation rates in asthma patients.^3^, ^4^, ^6^, ^7^ Here we demonstrate that the impairment of type I cDCs is associated with patient atopic status. The deficiency was most pronounced in patients with elevated serum IgE, indicative of those with more severe allergic disease. Numbers of type I cDCs were inversely associated with total serum IgE, and with house dust‐mite major allergen Der p1‐ and Der p2‐specific IgE. The underlying mechanism of this association is unclear, and requires further investigation, however, the novel observation that this DC subset expresses the high‐affinity IgE receptor at levels comparable to those observed on pDCs (Figure 6A), coupled with the knowledge that crosslinking this receptor on pDCs dramatically impairs their anti‐viral immune responses,^23^, ^24^ may provide a possible novel mechanism linking type I cDCs/IgE in the interplay between atopy and reduced anti‐viral responses.

The significant reduction of type I cDC numbers in the lower airways of asthma subjects at baseline was continued through a trend toward reduced numbers in asthma patients on both D3 and D8 during the course of the infection compared to their healthy counterparts. This observation did not reach statistical significance, however. There were also trends in both subject groups towards increased numbers of airway type I cDCs on both D3 and D8 during infection, compared to baseline, with greatest numbers being present on D8, suggesting that type I cDCs are actively recruited to the airway during respiratory viral infection. As little is understood about the mechanisms of DC recruitment to the airway, further studies are essential to understand the mechanisms underpinning DC recruitment to the airway and whether this is altered in asthma patients.

DC numbers in the airways have not previously been reported during respiratory viral infection in adults. Due to the more severe and prolonged symptomatic viral infections experienced in asthma,^6^, ^7^ supported by published data detailing increased virus loads^7^ and deficient anti‐viral immunity,^8^, ^9^ one could hypothesize that pDCs, a primary source of type I anti‐viral IFNs, would show reduced numbers in the airways in these patients. However, although numbers of pDCs were lower in asthma than in healthy control subjects at each time point studied, we did not identify any statistically significant differences in pDC numbers between subject groups, either at baseline or during infection. As with type I cDCs, we did not observe statistically significant pDC recruitment during infection, though numbers were again greatest on D8 in both subject groups. This failure to observe statistically significant recruitment could be due to the limited number of patients and time points studied.

We did however demonstrate, for the first time, that the expression of the specific IgE receptor FcϵRIα is increased on pDCs in the lower airways in asthma compared to healthy subjects, with median frequencies of FcϵRIα expressing pDCs of ~25% and 5%, at baseline respectively. Gill et al.^24^ have shown that FcεRIα on peripheral blood pDCs inversely correlated with their impaired production of IFN‐α in response to virus infection, and furthermore, that cross‐linking of FcεRIα (as would occur with allergen exposure in asthma) on peripheral blood pDCs ablates their ability to induce IFN‐α in response to virus infection. Furthermore, children on anti‐IgE therapy have a reduced rate of asthma exacerbations over time, and exhibit higher levels of virus‐induced IFN‐α when peripheral blood leukocytes are infected *ex vivo*.^25^ The increased expression of FcϵRIα on airway pDCs in asthma that we report herein strongly suggests that these cells are likely have deficient production of IFNs as well. Furthermore, this suggests another mechanism whereby allergen exposure can interact synergistically with virus infection in increasing exacerbation risk/severity (via allergen exposure ablating the ability of airway pDCs to respond to viral infection with IFN production, consequent upon the cross‐linking of FcεRIα on pDCs by allergen). Further *ex vivo* analyses would be needed to confirm this, however challenges relating to the low numbers of pDCs in the airways did not permit this during this study.

We also present the novel finding that type I cDCs in the lower airways express FcϵRIα. We identified a significant reduction in FcϵRIα expression on type I cDCs during the course of the infection in patients with asthma. Down‐regulation of expression has been documented in blood pDCs following *ex vivo* viral and CpG‐ODNstimulation,^24^, ^39^ and there was a trend towards reduction in the expression on airway pDCs during the course of the infection in patients with asthma in this study (Figure 6D), we can speculate this may suggest a shared mechanism of counter regulation between FcϵRIα and IFN production in both DC populations.

It is difficult to rule out the possibility of contamination with inflammatory DCs (infDC) in the type I cDC population, using commercially available markers, although this is unlikely at baseline. InfDCs can express both FcϵRIα and BDCA3,^40^ and a sub‐population which down‐regulate CD16 on activation may not have been removed using the lineage exclusion strategy (which eliminated cells expressing CD16). Subsequent to the design of this study, a comprehensive flow cytometric strategy to identify DCs has been published, which could be applied to human airway tissue in future studies to formally confirm this.^41^

This study has limitations, among which are the relatively small numbers of subjects studied in each group, which may have limited power to detect statistically significant differences between groups or between time points studied. Relatively small subject numbers are a necessary limitation of a complex invasive study in asthma patients with moderate disease severity, which was not well‐controlled at recruitment, and which involved them not only undergoing experimental respiratory virus infection, but also, for the first time, two bronchoscopies earlier (D3) and later (D8) during virus infection than the single time point of day 4 post‐infection previously studied.^7^, ^11^ Another limitation is that all asthma patients in this study were on ICS treatment for their disease, thus further studies would be required to determine the effect of corticosteroid usage on dendritic cell biology within the airways.

This study provides the first data detailing the complete enumeration of DC populations within the lower airways of asthma patients during steady‐state disease and further, provides the first documentation of the kinetics of airway DC migration following viral infection in man. Herein, we highlight deficiency in airway type I cDC numbers in asthma, which correlated with atopic status of the individual and was associated with reduced CD8^+^ cytotoxic T cell recruitment and worse clinical, virologic and pathologic outcomes during infection. Further this study provides a basis for the understanding of DC kinetics in the airways. More work is needed to address the questions raised, however, the expression of FcϵRIα on type I cDCs may provide a link as to why we see greater impairment in the most severely atopic patients.

## CONFLICT OF INTEREST

SLJ has received personal fees from Virtus Respiratory Research Ltd, Myelo Therapeutics GmbH, Bayer, Synairgen, Novartis, Boehringer Ingelheim, Chiesi, Gerson Lehrman Group, resTORbio, Bioforce, Materia Medical Holdings, PrepBio Pharma, Pulmotect, Virion Health, Lallemand Pharma and AstraZeneca. SLJ is an author on patents on the use of inhaled interferons for exacerbations of airway disease.

## AUTHOR CONTRIBUTIONS

A.C. performed bronchoalveolar lavage processing, flow cytometric staining and analysis, differential cell counts and data analysis. J.D. performed the clinical aspects of the study. E.W. performed post‐infection bronchoscopies. M.P. performed BAL FACS sorting. N.U. conducted ImmunoCAP serum IgE measurement. I.R.J conducted flow cytometric analysis of CD8+ T cells. M‐B.T‐T. and J.d‐R. assisted with screening volunteers and sampling. S.L.J. and D.J.J. supervised clinical aspects of the study. R.P.W. and M.P. advised on flow cytometric analysis. R.P.W. and M.R.E. supervised laboratory processing and analysis.

## Supporting information

Fig S1

Fig S2

Table S1

Table S2

Table S3

Table S4

Method S1
