# Supplementary figures and images for: Type I conventional dendritic cells relate to disease severity in virus‐induced asthma exacerbations
Source: Clin Exp Allergy. 2022 Mar 3;52(4):550–60. doi: 10.1111/cea.14116 (PMC9310571; doi:10.1111/cea.14116)

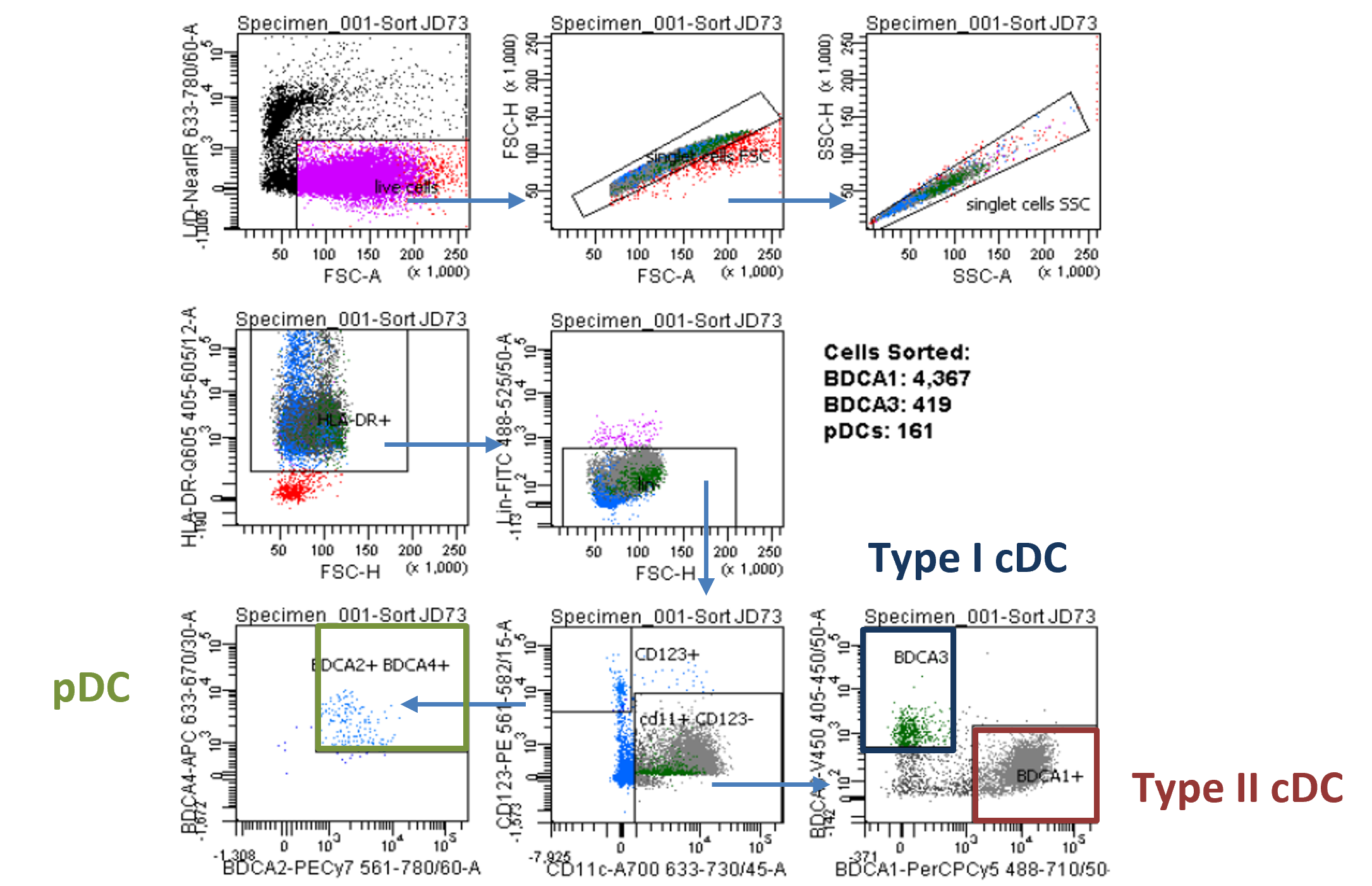

Supplement: Supplementary file 1 — Fig S1 [file CEA-52-550-s005.png]

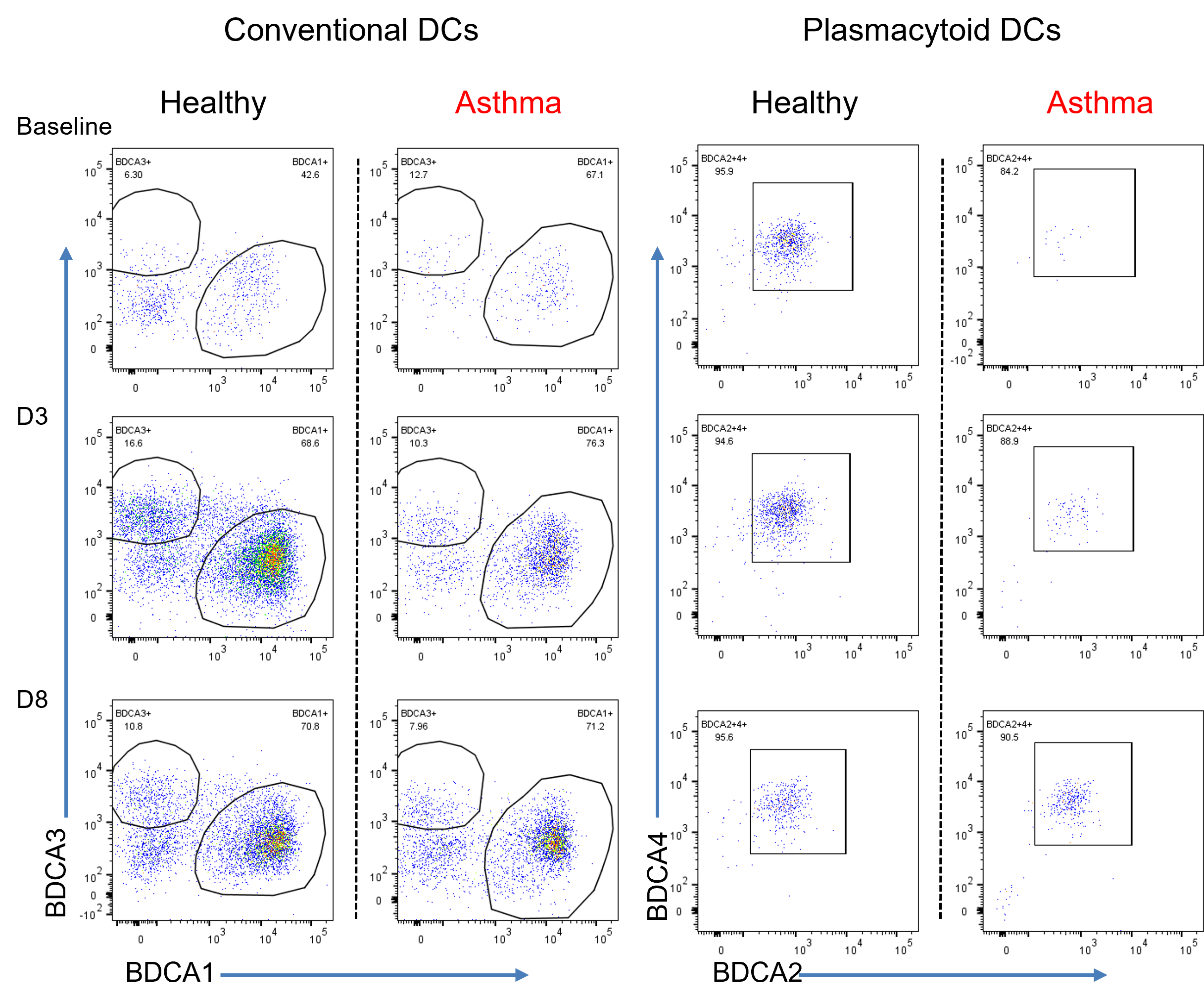

Supplement: Supplementary file 2 — Fig S2 [file CEA-52-550-s004.png]
