# Supplementary material for: Type I conventional dendritic cells relate to disease severity in virus‐induced asthma exacerbations
Source: Clin Exp Allergy. 2022 Mar 3;52(4):550–60. doi: 10.1111/cea.14116 (PMC9310571; doi:10.1111/cea.14116)
Supplement: Supplementary file 3 — Table S1 [file CEA-52-550-s003.docx]

| **Atopic Asthma Subject Inclusion Criteria** | |
| --- | --- |
| Age 18-55 years | Histamine PC_20_ <8ug/ml (or <12ug/ml and bronchodilator response ≥12%) |
| Clinical diagnosis of asthma | Daily ICS (daily dose ≥400mcg fluticasone or equivalent) or ICS and LABA combination inhaler |
| Positive skin prick test to a panel of ten aeroallergens  RV-16 neutralising antibody seronegative | ACQ score > 0.75 |
| **Atopic Asthma Subject Exclusion Criteria** | |
| Current use of anti-histamines, nasal steroids, LTRA or tiotropium | History of clinically relevant systemic disease or respiratory disease (other than asthma) |
| Current symptoms of rhinitis | OCS treatment in the previous 3 months |
| Smoking history in the past 6 months | Pregnant or breastfeeding women |
| **Healthy Subject Inclusion Criteria** | |
| Age 18-55 years  RV-16 neutralising antibody seronegative | Histamine PC_20_ >8ug/ml and bronchodilator response <12% |
| **Healthy Subject Exclusion Criteria** | |
| Smoking history in the past 6 months | History of respiratory or significant systemic disease |
| Positive skin prick test | Use of ICS or OCS in the previous 3 months |
| History or current symptoms of atopic disease such as allergic rhinitis, asthma or eczema | Current use of LABA, nasal spray, anti-histamine, LTRA or tiotropium |
| Shortness of breath at screening | Pregnant or breastfeeding women |
