# Supplementary material for: Type I conventional dendritic cells relate to disease severity in virus‐induced asthma exacerbations
Source: Clin Exp Allergy. 2022 Mar 3;52(4):550–60. doi: 10.1111/cea.14116 (PMC9310571; doi:10.1111/cea.14116)
Supplement: Supplementary file 4 — Table S2 [file CEA-52-550-s006.docx]

| **Procedures** | **Time point (Day)** | | | | | | | | | | | | | |  |
| --- | --- | --- | --- | --- | --- | --- | --- | --- | --- | --- | --- | --- | --- | --- | --- |
|  | -15 | -14 | 0 | 1 | 2 | 3 | 4 | 5 | 6 | 7 | 8 | 11 | 15 | 42 |  |
| Virus Inoculation |  |  | **x** |  |  |  |  |  |  |  |  |  |  |  |  |
| Bronchoscopy:  BAL |  | **X** |  |  |  | **X** |  |  |  |  | **X** |  |  |  |  |
| Nasal lavage |  | **X** | **X** | **X** | **X** | **X** | **X** | **X** | **X** | **X** | **X** | **X** | **X** | **X** |  |
| Clinic Spirometry |  | **X** |  |  | **X** |  | **X** | **X** |  | **X** | **X** | **X** | **X** | **X** |  |
| Histamine  Challenge | **X** |  |  |  |  |  |  |  |  |  |  |  |  |  |  |
| Symptom Diaries | Daily at home during study period | | | | | | | | | | | | | | |
