# Supplementary material for: Type I conventional dendritic cells relate to disease severity in virus‐induced asthma exacerbations
Source: Clin Exp Allergy. 2022 Mar 3;52(4):550–60. doi: 10.1111/cea.14116 (PMC9310571; doi:10.1111/cea.14116)
Supplement: Supplementary file 5 — Table S3 [file CEA-52-550-s001.docx]

| **Antibody** | **Fluorochrome** | **Supplier** | **Clone number** |
| --- | --- | --- | --- |
| **BAL DC Sort Panel** | | | |
| Lineage Cocktail | FITC | eBioscience | * |
| FcεRIα | BV510 | Biolegend | AER-37 (CRA-1) |
| HLA-DR | QDOT605 | Invitrogen | TÜ36 |
| CD11c | AlexaFluor 700 | eBioscience | 3.9 |
| CD123 | PE | eBioscience | 6H6 |
| BDCA1 | PerCP eFluor 710 | eBioscience | L161 |
| BDCA2 | PE-Cy7 | eBioscience | 201a |
| BDCA3 (CD141) | VioBlue | Miltenyi | AD5-14H12 |
| BDCA4 (CD304) | APC | Miltenyi | AD5-17F6 |
| Live/Dead Stain | Near IR | Life Technologies |  |
| **BAL T cell Sort Panel** | | | |
| CD3 | APC | BD Biosciences | SP34-2 |
| CD8 | APC-Cy7 | Biolegend | HIT8A |
| CD4 | BV421 | Biolegend | OKT4 |
| Live/Dead Stain |  | Biolegend |  |
