# Supplementary material for: Type I conventional dendritic cells relate to disease severity in virus‐induced asthma exacerbations
Source: Clin Exp Allergy. 2022 Mar 3;52(4):550–60. doi: 10.1111/cea.14116 (PMC9310571; doi:10.1111/cea.14116)
Supplement: Supplementary file 6 — Table S4 [file CEA-52-550-s007.docx]

|  |  | **Healthy** | **Asthma** |
| --- | --- | --- | --- |
| Figure 3A | Spearman r | -0.1888 | 0.05455 |
|  | P value | 0.5577 | 0.8812 |
| Figure 4A | Spearman r | -0.4858 | -0.5376 |
|  | P value | 0.1126 | 0.0914 |
| Figure 4B | Spearman r | 0.2818 | 0.6000 |
|  | P value | 0.4023 | 0.0734 |
| Figure 4C | Spearman r | -0.1926 | -0.3918 |
|  | P value | 0.5459 | 0.2320 |
| Figure 4D | Spearman r | -0.3521 | -0.2661 |
|  | P value | 0.2619 | 0.4291 |
