# Supplementary material for: Type I conventional dendritic cells relate to disease severity in virus‐induced asthma exacerbations
Source: Clin Exp Allergy. 2022 Mar 3;52(4):550–60. doi: 10.1111/cea.14116 (PMC9310571; doi:10.1111/cea.14116)
Supplement: Supplementary file 7 — Method S1 [file CEA-52-550-s002.docx]

**Type I Conventional Dendritic Cells Relate to Disease Severity in Virus-Induced Asthma Exacerbations**

Aoife Cameron^1,2^, Jaideep Dhariwal^1,2^_,_ Nadine Upton^2,4^_,_ Ismael Ranz Jimenez^2,4^_,_ Malte Paulsen^3^, Ernie Wong^1,2^, Maria-Belen Trujillo-Torralbo^1,2^, Ajerico del Rosario^1,2^, David J Jackson^1,2,5^, Michael R Edwards^1,2^, Sebastian L Johnston^1,2*^ and Ross P Walton^1,2*^ on behalf of the MRC-GSK strategic alliance consortium
*These authors contributed equally to this work

Online Supplement

**Supplementary Methods**

**Flow Cytometer Specifications**

PMT voltages were adjusted after standardized CST checks, minimizing the spectral overlap to increase data precision. BAL cells were sorted using a Becton Dickinson AriaIIIu equipped with 50mW 405nm, 50mW 488nm, 50mW 561nm, 20mW 633nm lasers and a ND1.5 filter in front of the FSC photodiode. A nozzle size of 100um was used and the corresponding BD FACSFlow sheath pressure of 20psi, matched with a transducer frequency of 29.1kHz. Input pressure was adjusted to ensure that every 5^th^ to 6^th^ drop was populated by an event.

**Author Appendix**

MRC-GSK strategic alliance consortium list:

Sebastian L Johnston^1,2^, Roberto Solari^1,2^, Michael R Edwards^1,2^, Paul Lavender^2,4^, Ross P Walton^1,2^, Hannah Gould^2,4^, David Cousins^2,4,5^, Antoon J. van Oosterhout^3^, Jaideep Dhariwal^1,2^, Aoife Cameron^1,2^, Nathan W Bartlett^1,2^, Patrick Mallia^1,2^, David J Jackson^1,2,6^, Maria-Belen Trujillo-Torralbo^1,2^, Jerico del Rosario^1,2^, Janet L. Smith^3^, Matthew J. Edwards^3^, Karen Affleck^3^, Nil Turan Jurdzinski^3^, Veronique Birault^3^, Peter McErlean^2,4^, Yu-Chang Wu^2,4^, Nadine Upton^2,4^, Ismael Ranz Jimenez^2,4^.

Author Affiliations:

1. National Heart and Lung Institute, Imperial College London, London, UK

2. MRC and Asthma UK Centre in Allergic Mechanism of Asthma, London, UK

3. GlaxoSmithKline, Allergic Inflammation Discovery Performance Unit, Respiratory Therapy Area, Stevenage, UK

4. Department of Respiratory Medicine & Allergy, King’s College London, London, UK
5. NIHR Respiratory Biomedical Research Unit, Department of Infection, Immunity & Inflammation, Leicester Institute for Lung Health, University of Leicester, Leicester, UK
6. Guy's and St Thomas' NHS Trust, London, UK

**Figure Legends**

**Figure E1. Flow cytometry gating strategy to isolate lower airway dendritic cell populations.** BAL fluid was collected during bronchoscopy and following a primary sort to enrich lineage^-^ HLA-DR^+^ cells, a secondary sort of HLA-DR^+^ lineage^-^ cells was carried out. Live, single, HLA-DR^+^ lineage^-^ were sorted in to type I cDCs (CD11c^+^ BDCA3^+^), type II cDCs (CD11c^+^ BDCA1^+^) and pDCs (CD123^+^ BDCA2^+^ BDCA4^+^). Abbreviations: DC dendritic cell, BAL bronchoalveolar lavage, BDCA blood dendritic cell antigen.

**Figure E2. Representative DC populations in the lower airways at baseline and following experimental RV-16 infection.** BAL was collected at baseline, D3 and D8 following experimental RV-16 infection in atopic asthmatic and healthy control subjects. DC populations were sorted from BAL and representative flow cytometry gating plots from an asthmatic patient and a healthy control are shown, depicting conventional DCs (HLA-DR^+^ lineage^-^, CD11c^+^ CD123^-^) type I (BDCA3^+^) and type II (BDCA2^+^), and pDCs (HLA-DR^+^ lineage^-^, CD123^+^ CD11c^-^, BDCA2^+^ BDCA4^+^). Abbreviations: DC dendritic cell, BAL bronchoalveolar lavage, BDCA blood dendritic cell antigen, RV rhinovirus.

**Tables**

| **Atopic Asthma Subject Inclusion Criteria** | |
| --- | --- |
| Age 18-55 years | Histamine PC_20_ <8ug/ml (or <12ug/ml and bronchodilator response ≥12%) |
| Clinical diagnosis of asthma | Daily ICS (daily dose ≥400mcg fluticasone or equivalent) or ICS and LABA combination inhaler |
| Positive skin prick test to a panel of ten aeroallergens  RV-16 neutralising antibody seronegative | ACQ score > 0.75 |
| **Atopic Asthma Subject Exclusion Criteria** | |
| Current use of anti-histamines, nasal steroids, LTRA or tiotropium | History of clinically relevant systemic disease or respiratory disease (other than asthma) |
| Current symptoms of rhinitis | OCS treatment in the previous 3 months |
| Smoking history in the past 6 months | Pregnant or breastfeeding women |
| **Healthy Subject Inclusion Criteria** | |
| Age 18-55 years  RV-16 neutralising antibody seronegative | Histamine PC_20_ >8ug/ml and bronchodilator response <12% |
| **Healthy Subject Exclusion Criteria** | |
| Smoking history in the past 6 months | History of respiratory or significant systemic disease |
| Positive skin prick test | Use of ICS or OCS in the previous 3 months |
| History or current symptoms of atopic disease such as allergic rhinitis, asthma or eczema | Current use of LABA, nasal spray, anti-histamine, LTRA or tiotropium |
| Shortness of breath at screening | Pregnant or breastfeeding women |

**Table E1: Study inclusion and exclusion criteria.** Abbreviations: ACQ asthma control questionnaire, ICS inhaled corticosteroid, LABA long-acting β_2_ agonist, LTRA leukotriene receptor antagonist, OCS oral corticosteroid, PC_20_ concentration of histamine required to reduce FEV_1_ by 20%.

| **Procedures** | **Time point (Day)** | | | | | | | | | | | | | |  |
| --- | --- | --- | --- | --- | --- | --- | --- | --- | --- | --- | --- | --- | --- | --- | --- |
|  | -15 | -14 | 0 | 1 | 2 | 3 | 4 | 5 | 6 | 7 | 8 | 11 | 15 | 42 |  |
| Virus Inoculation |  |  | **x** |  |  |  |  |  |  |  |  |  |  |  |  |
| Bronchoscopy:  BAL |  | **X** |  |  |  | **X** |  |  |  |  | **X** |  |  |  |  |
| Nasal lavage |  | **X** | **X** | **X** | **X** | **X** | **X** | **X** | **X** | **X** | **X** | **X** | **X** | **X** |  |
| Clinic Spirometry |  | **X** |  |  | **X** |  | **X** | **X** |  | **X** | **X** | **X** | **X** | **X** |  |
| Histamine  Challenge | **X** |  |  |  |  |  |  |  |  |  |  |  |  |  |  |
| Symptom Diaries | Daily at home during study period | | | | | | | | | | | | | | |

**Table E2. Summary of experimental RV-16 challenge study visits and procedures.**

| **Antibody** | **Fluorochrome** | **Supplier** | **Clone number** |
| --- | --- | --- | --- |
| **BAL DC Sort Panel** | | | |
| Lineage Cocktail | FITC | eBioscience | * |
| FcεRIα | BV510 | Biolegend | AER-37 (CRA-1) |
| HLA-DR | QDOT605 | Invitrogen | TÜ36 |
| CD11c | AlexaFluor 700 | eBioscience | 3.9 |
| CD123 | PE | eBioscience | 6H6 |
| BDCA1 | PerCP eFluor 710 | eBioscience | L161 |
| BDCA2 | PE-Cy7 | eBioscience | 201a |
| BDCA3 (CD141) | VioBlue | Miltenyi | AD5-14H12 |
| BDCA4 (CD304) | APC | Miltenyi | AD5-17F6 |
| Live/Dead Stain | Near IR | Life Technologies |  |
| **BAL T cell Sort Panel** | | | |
| CD3 | APC | BD Biosciences | SP34-2 |
| CD8 | APC-Cy7 | Biolegend | HIT8A |
| CD4 | BV421 | Biolegend | OKT4 |
| Live/Dead Stain |  | Biolegend |  |

**Table E3: Antibodies Used for Flow Cytometry and FACS**. *Lineage cocktail contained the following antibodies: CD2 (RPA-2.10), CD3 (OKT3), CD14 (61D3), CD16 (CB16), CD19 (HIB19), CD56 (CB56), CD235a (HIR2).

|  |  | **Healthy** | **Asthma** |
| --- | --- | --- | --- |
| Figure 3A | Spearman r | -0.1888 | 0.05455 |
|  | P value | 0.5577 | 0.8812 |
| Figure 4A | Spearman r | -0.4858 | -0.5376 |
|  | P value | 0.1126 | 0.0914 |
| Figure 4B | Spearman r | 0.2818 | 0.6000 |
|  | P value | 0.4023 | 0.0734 |
| Figure 4C | Spearman r | -0.1926 | -0.3918 |
|  | P value | 0.5459 | 0.2320 |
| Figure 4D | Spearman r | -0.3521 | -0.2661 |
|  | P value | 0.2619 | 0.4291 |

**Table E4: Associations from Figures 3(A) and 4 shown as asthmatic and healthy groups separately.** Figure 3 (A) Numbers of type I cDCs in BAL at baseline were correlated with baseline total serum IgE. Figure 4 Numbers of type I cDCs in BAL were correlated with eosinophil numbers in the BAL at D3 post RV-16 infection (A), change from baseline in FEV1 at D8 (B) and nasal lavage virus load at D4 (C) and D5 (D).
